# PGC‐1α overexpression increases transcription factor EB nuclear localization and lysosome abundance in dystrophin‐deficient skeletal muscle

**DOI:** 10.14814/phy2.14383

**Published:** 2020-02-28

**Authors:** Hannah R. Spaulding, Amanda K. Ludwig, Katrin Hollinger, Matthew B. Hudson, Joshua T. Selsby

**Affiliations:** ^1^ Department of Animal Science Iowa State University Ames IA USA; ^2^ Department of Biological Sciences Purdue University West Lafayette IN USA; ^3^ Department of Kinesiology and Applied Physiology University of Delaware Newark DE USA

**Keywords:** autophagy, DMD, dystrophin, mdx, TFEB

## Abstract

Duchenne muscular dystrophy (DMD) is caused by the absence of functional dystrophin protein and results in progressive muscle wasting. Dystrophin deficiency leads to a host of dysfunctional cellular processes including impaired autophagy. Autophagic dysfunction appears to be due, at least in part, to decreased lysosomal abundance mediated by decreased nuclear localization of transcription factor EB (TFEB), a transcription factor responsible for lysosomal biogenesis. PGC‐1α overexpression decreased disease severity in dystrophin‐deficient skeletal muscle and increased PGC‐1α has been linked to TFEB activation in healthy muscle. The purpose of this study was to determine the extent to which PGC‐1α overexpression increased nuclear TFEB localization, increased lysosome abundance, and increased autophagosome degradation. We hypothesized that overexpression of PGC‐1α would drive TFEB nuclear translocation, increase lysosome biogenesis, and improve autophagosome degradation. To address this hypothesis, we delivered PGC‐1α via adeno‐associated virus (AAV) vector injected into the right limb of 3‐week‐old mdx mice and the contralateral limbs received a sham injection. At 6 weeks of age, this approach increased PGC‐1α transcript by 60‐fold and increased TFEB nuclear localization in gastrocnemii from PGC‐1α treated limbs by twofold compared to contralateral controls. Furthermore, lamp2, a marker of lysosome abundance, was significantly elevated in muscles from limbs overexpressing PGC‐1α. Lastly, increased LC3II and similar p62 in PGC‐1α overexpressing‐limbs compared to contralateral limbs are supportive of increased degradation of autophagosomes. These data provide mechanistic insight into PGC‐1α‐mediated benefits to dystrophin‐deficient muscle, such that increased TFEB nuclear localization in dystrophin‐deficient muscle leads to increased lysosome biogenesis and autophagy.

## Graphical abstract

Degradation of autophagosomes appears to be impaired in dystrophic skeletal muscle in part due to reduced lysosome abundance. We found that PGC‐1alpha mediated TFEB nuclear translocation, increased lysosome abundance, and degradation of autophagosomes.

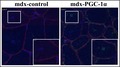

## 1 INTRODUCTION

Duchenne muscular dystrophy (DMD) is a devastating muscle‐wasting disease affecting 1:3,500–5,000 boys (Mah et al., 2014). This progressive disease is typically diagnosed from 3–5 years of age due to muscle weakness and locomotor dysfunction, advances to wheelchair use for mobility by their early teens, and culminates in death by cardiac or respiratory failure in their mid to late twenties. DMD is caused by the absence of functional dystrophin protein and results in various cellular dysfunctions such as membrane instability, mitochondrial dysfunction, oxidative stress, calcium mishandling, and dysregulation of autophagy (Alderton and Steinhardt, 2000; Bibee et al., 2014; De Palma et al., 2012; Godin et al., 2012; Pal et al., 2014; Spaulding et al., 2018). Autophagy is a process by which cellular components tagged for degradation are collected within autophagosomes, then fused with lysosomes to degrade the autophagosome cargo (Mizushima, Ohsumi, & Yoshimori, 2002; Ryter, Cloonan, & Choi, 2013). In dystrophin‐deficient skeletal muscle, autophagosome degradation is blunted, resulting in autophagosome accumulation and subsequent escape of autophagosomes into the extracellular space (Spaulding et al., 2018). In this same study, lysosome abundance was decreased in dystrophin‐deficient skeletal muscle (Spaulding et al., 2018), therefore, it seems likely that reduced lysosomal abundance, at least in part, contributes to blunted degradation of autophagosomes.

Lysosome biogenesis is regulated by transcription factor EB (TFEB) (Sardiello et al., 2009; Settembre et al., 2011), which is translocated to the nucleus to promote transcription of lysosomal and autophagy‐related genes. Nuclear TFEB localization is regulated by phosphorylation in which dephosphorylation of TFEB by calcineurin at Ser142 (Medina et al., 2015) promotes nuclear translocation. Conversely, phosphorylation by mammalian target of rapamycin (mTOR), protein kinase B (AKT), or extracellular signal‐regulated protein kinase (ERK1/2) sequesters TFEB in the cytosol (Martina, Chen, Gucek, & Puertollano, 2012; Palmieri et al., 2017; Settembre et al., 2011). We have previously demonstrated TFEB nuclear localization is decreased in dystrophin‐deficient muscle compared to healthy muscle (Spaulding et al., 2018. In healthy muscle, TFEB abundance was decreased in peroxisome proliferator‐activated receptor‐gamma coactivator 1‐alpha (PGC‐1α) knockout mice (Erlich, Brownlee, Beyfuss, & Hood, 2018; Vainshtein, Desjardins, Armani, Sandri, & Hood, 2015); conversely, TFEB nuclear localization and transcription were increased with exercise suggesting a PGC‐1α‐dependent mechanism and indicative of a relationship between PGC‐1α and TFEB (Erlich et al., 2018). We and others have previously found that PGC‐1α overexpression decreased disease‐related injury though attributed protection largely to increased utrophin expression associated with a type I fiber shift (Handschin et al., 2007; Selsby, Morine, Pendrak, Barton, & Sweeney, 2012). The extent to which PGC‐1α may also increase TFEB nuclear localization, lysosomal content, and degradation of autophagosomes has not been previously considered in this context. Given that PGC‐1α plays a role in TFEB regulation and localization, we hypothesized that overexpression of PGC‐1α would increase TFEB nuclear localization and ultimately increase lysosome abundance and restore autophagosome degradation in dystrophin‐deficient skeletal muscle.

## 2 METHODS

### 2.1 Animal treatment

The Institutional Animal Care and Use Committee at Iowa State University approved all procedures. A detailed description of animal treatments and a demonstration of PGC‐1α‐mediated rescue of dystrophic soleus muscles has been previously published (Hollinger et al., 2013). Briefly, at three‐weeks of age, 1x10^11^ genome copies of an adeno‐associated viral (AAV; serotype 6) vector driving expression of PGC‐1α transcript was injected into the right triceps surae group of male and female mdx mice in a single 50 μl dose, while the contralateral limb was injected with an empty capsid (*n* = 8 mice/group). Generally, 5–6 sample pairs were randomly selected for each experiment and this is explicitly stated in figure legends. We have previously used this vector to demonstrate disease prevention and rescue (Hollinger et al., 2013; Hollinger & Selsby, 2015; Selsby et al., 2012). At 6 weeks of age, we sacrificed mice by cervical dislocation and the gastrocnemii were harvested. Gastrocnemii designated for western blotting and qPCR were snap‐frozen in liquid nitrogen, and gastrocnemii for immunofluorescence were frozen in isopentane and embedded in optimal cutting temperature compound (OCT). Data from these mice have been previously published establishing PGC‐1α‐mediated histological rescue of treated muscle (Hollinger et al., 2013).

### 2.2 qPCR

Quantitative PCR was used to confirm PGC‐1α transcript overexpression and was done as previously described (Selsby, Ballmann, Spaulding, Ross, & Quindry, 2016). Briefly, gastrocnemii were powdered on dry ice and RNA was extracted using TriZol (15596018, ThermoScientific) and RNeasy purification kit (Qiagen) according to manufacturer instructions. cDNA was reverse transcribed using QuantiTect Reverse Transcriptase Kit (205310, Qiagen), as described by the manufactures except random hexamers (51‐0118‐01, IDT Premade Primers) were used instead of the RT primer mix provided (Qiagen). The PGC‐1α transcript primer set was as follows: Forward – 5'atgtgtcgccttcttgctct‐3' Reverse – 5'‐atctactgcctggggacctt‐3'. PGC‐1α transcript expression was normalized to *18s* (Forward – 5'‐ctctagataacctcgggccg‐3' Reverse – 5'‐gtcgggagtgggtaatttgc‐3'). In our hands *18s* was highly stable in sample pairs and between groups (mean Control CT – 16.2; mean PGC‐1α CT – 16.3; *p* > .9) and avoids potential treatment effects on other housekeeping genes (Hollinger et al., 2013). Delta (Δ) CT values were calculated by subtracting the 18s CT value from the PGC‐1α mRNA CT value. These values were used for statistical comparisons. ΔΔCT was calculated as the difference in ΔCT of each pair of samples. Data are presented as a fold‐change as calculated from ΔΔCT.

### 2.3 Western blot

Western blotting was performed as previously described (Spaulding, Ballmann, Quindry, & Selsby, 2016). Briefly, whole homogenate, nuclear protein fractions, and cytoplasmic protein fractions were extracted from powdered gastrocnemius muscles. Total protein was extracted using lysis buffer (1% Triton‐x‐100, 50 mM HEPESS, 150 mM NaCl, 10% Glycerol, 50 mM NaF, 2 mM EDTA, 0.1% SDS, pH 7.5). Additionally, the nuclear protein fraction was separated from the cytoplasmic fraction using a NE‐PER™ Nuclear and Cytoplasmic Extraction Reagents (78833, ThermoScientific). Total, nuclear and cytoplasmic protein abundances were measured by Pierce BCA Protein Assay Kit (23225, ThermoScientific) and diluted in 2X Laemmli buffer (161‐0737, Bio‐Rad). For experiments using whole homogenate 40 μg and for cytoplasmic and nuclear fraction experiments 12 μg protein was loaded into 4%–20% gels (58545, Lonza), separated by gel electrophoresis and transferred to a nitrocellulose membrane (1620112, Bio‐Rad). Membranes were incubated with Ponceau S (PS) stain and the total optical density of each lane was quantified to verify equal loading. This approach eliminates potential impacts of treatment on commonly used single‐protein loading controls (Aldridge, Podrebarac, Greenough, & Weiler, 2008; Gardan‐Salmon, Dixon, Lonergan, & Selsby, 2011; Moritz, 2017). Membranes were washed in 1X tris buffered saline with 0.2% Tween20 (1X TBST) and blocked with 5% milk in 1X TBST for 1 hr. Primary antibody (Table 1) was applied to the membrane and rocked overnight at 4ºC. Once primary antibody was removed, the membrane was washed in 1X TBST for 10 min, three times. Following washes, anti‐rabbit IgG HRP‐linked secondary antibody (7074, Cell Signaling) or anti‐rat IgG HRP‐linked secondary antibody (7077, Cell Signaling) was applied to the membrane for 1 hr at room temperature. After 1 hr the membrane was washed again for 10 min, three times. Lastly, Clarity™ Western enhanced chemiluminescence (1705060S, Bio‐Rad) was applied to the membrane for 7 min, then developed in a dark room. We used Carestream software to objectively quantify the optical density of each band using the automated band‐find function where possible to limit unconscious bias that may occur. The local background subtraction feature was used to account for variations in background intensity across each film and we confirmed that all images were free of saturated pixels before quantification. Bands were identified on the basis of molecular weight using validated antibodies.

**Table 1:** Western blot and immunofluorescence antibody details including protein name, primary and secondary dilations, and product number. Secondary antibodies used were specific to the host species, which is also indicated in this table along with the product number, and secondary antibody details can be found in the methods sections

Western blot antibodies			
Protein	Primary dilution	Secondary dilution	Company & Product number (host)
Autophagy related (ATG) 5/12	1:1,000 1X TBST	1:2000 1X TBST	Cell signaling 4180 (rabbit)
Beclin‐1	1:500 5% milk	1:2000 5% milk	Cell signaling 3495 (rabbit)
Phospho (p) beclin‐1 (Ser93)	1:1,000 5% milk	1:1,000 1X TBST	Cell signaling 14717 (rabbit)
Light chain 3 (LC3)	1:500 5% milk	1:2000 5% milk	Cell signaling 12741 (rabbit)
Lysosomal‐associated membrane protein 2 (lamp2)	1:1,000 1X TBST	1:2000 5% milk	Abcam ab13524 (rat)
Phosphoinositide 3 kinases (PI3K) class III	1:1,000 5% milk	1:2000 5% milk	Cell signaling 3358 (rabbit)
Sequestosome 1 (SQSTM1, p62)	1:500 5% milk	1:1,000 5% milk	Abcam ab109012 (rabbit)
Transcription factor EB (TFEB)	1:1,000 1% milk	1:1,000 1X TBST	Bethyl laboratories A303‐673A‐*M* (rabbit)
Unc‐51 Like autophagy activating kinase 1 (ULK)	1:1,000 1X TBST	1:2000 1X TBST	Cell signaling 8054 (rabbit)
p‐ULK (Ser555)	1:500 1X TBST	1:1,000 1X TBST	Cell signaling 5869 (rabbit)
Immunofluorescence antibodies			
laminin beta‐1	1:100 5% BSA	1:200 5% BSA	Thermofisher MA5‐14657 (rat)
Lysosomal‐associated membrane protein 2 (lamp2)	1:100 5% BSA	1:100 5% BSA	Abcam ab13524 (rat)
Transcription factor EB (TFEB)	1:50 5% BSA	1:500 5% BSA	Bethyl laboratories A303‐673A‐*M* (rabbit)

Immunofluorescence. We performed Immunofluorescence as previously described (Spaulding et al., 2018). Briefly, muscle sections were blocked for 45 min in blocking solution (5% goat serum, 5% bovine serum albumin, 1% DMSO in 2X PBS). We applied primary antibody (Table 1) to the slides and placed the slides at 4°C overnight, then washed the slides for 10 min in 1X phosphate‐buffered saline (PBS) three times. Corresponding secondary antibody was applied to sections for 1 hr at room temperature in the dark (anti‐ rabbit IgG AlexaFluor 488 [Cell Signaling, 4412] or goat anti‐rat IgG rhodamine conjugated [Thermo Scientific, 31680]) and then slides were washed in 1X PBS for 10 min, three times. Finally, sections were treated with SlowFade™ Gold Antifade Mountant with DAPI (S36938, ThermoScientific) and sealed with nail polish. Sections were imaged on a QICAM 12‐bit Mono Fast 1394 Cooled (QIC‐F‐M‐12‐C, QIMAGING) camera attached to a Leica microscope at 40x, and confocal images were taken at 63x on a similar Leica microscope.

Statistics. Data were statistically assessed using paired *t* test (PRISM). Data are reported as mean ± SEM. We consider *p*‐values less than or equal to .05 to be significant.

## 3 RESULTS

### 3.1 PGC‐1α overexpression increased nuclear TFEB abundance

**Figure 1:**
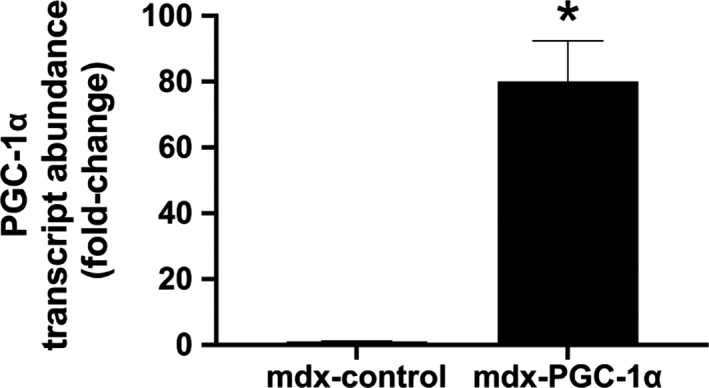
PGC‐1α transcript expression increased following gene delivery. PGC‐1α transcript abundance was normalized to 18S and confirmed overexpression of PGC‐1α in AAV‐treated gastrocnemii (*n* = 8/group). Quantification by delta delta CT is explained in the Methods section, * = significantly different from mdx‐control by paired sample *t* test, *p* < .05

**Figure 2:**
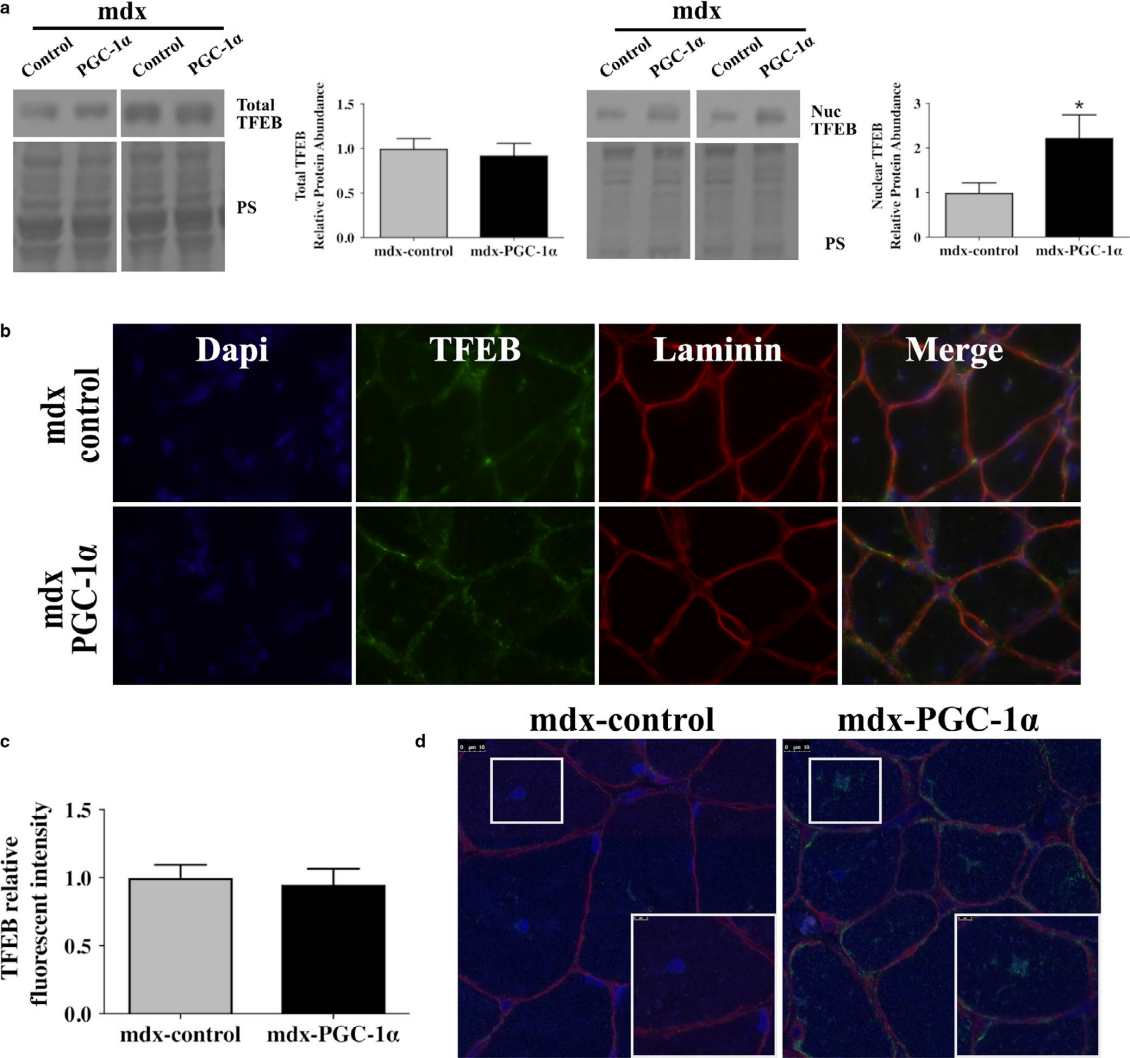
PGC‐1α overexpression increased nuclear TFEB. (a) Total TFEB protein abundance was similar between groups regardless of treatment, (*n* = 6/group). Nuclear TFEB abundance significantly increased with PGC‐1α overexpression, (*n* = 6/group). Ponceau stain (PS) was used as a total protein loading control. (b) mdx‐control and mdx‐PGC‐1α‐overexpressing gastrocnemii were probed for TFEB (green), membrane marker, Laminin (red), and nuclei (dapi, blue) using immunofluorescence. (c) Immunofluorescence quantification confirms that total TFEB abundance was similar between mdx‐control and mdx‐ PGC‐1α, (*n* = 5/group) (d) Representative confocal images from C at 63X with 5x zoomed insets showing the increased nuclear localization of TFEB with PGC‐1α overexpression. * = significantly different from mdx‐control by paired sample *t* test, *p* < .05

Effective delivery of the PGC‐1α transgene was confirmed using qPCR (Figure 1). Consistent with our previous experiments (Hollinger et al., 2013; Hollinger & Selsby, 2015; Selsby et al., 2012), PGC‐1α transcript was increased approximately 80‐fold. Of note, the 80‐fold elevation observed in this investigation was greater than the 10‐fold elevation observed in soleus muscles obtained from the same mice (Hollinger et al., 2013). It is likely that the differing fiber types of the muscles and/or that the gastrocnemius potentially received a greater content of the injected volume (and hence, viral particles) contributed to this difference. PGC‐1α overexpression resulted in an approximate 6 mg reduction (*p* < .05) in muscle mass compared to control (Control: ~103 ± 4; PGC‐1α: 97 ± 4 mg), which we previously reported (Hollinger et al., 2013). To assess the effect of PGC‐1α overexpression on TFEB abundance, TFEB protein abundance was measured in whole homogenate by Western blot. Total TFEB abundance was similar between groups (Figure 2a). To determine the effect of PGC‐1α overexpression on TFEB nuclear localization, TFEB protein abundance was assessed in nuclear protein fractions. PGC‐1α overexpression increased nuclear TFEB abundance 2.2‐fold compared to untreated limbs (Figure 2a). In confirmatory experiments using an immunofluorescent approach it was visually apparent that total TFEB was similar between groups, which was further supported by objective quantification of total fluorescence (Figure 2b,C). Subjective evaluation of confocal images were supportive of nuclear translocation of TFEB in PGC‐1α‐overexpressing limbs compared to contralateral controls (Figure 2d).

### 3.2 PGC‐1α overexpression increased lysosome abundance and markers of autophagosome degradation

**Figure 3:**
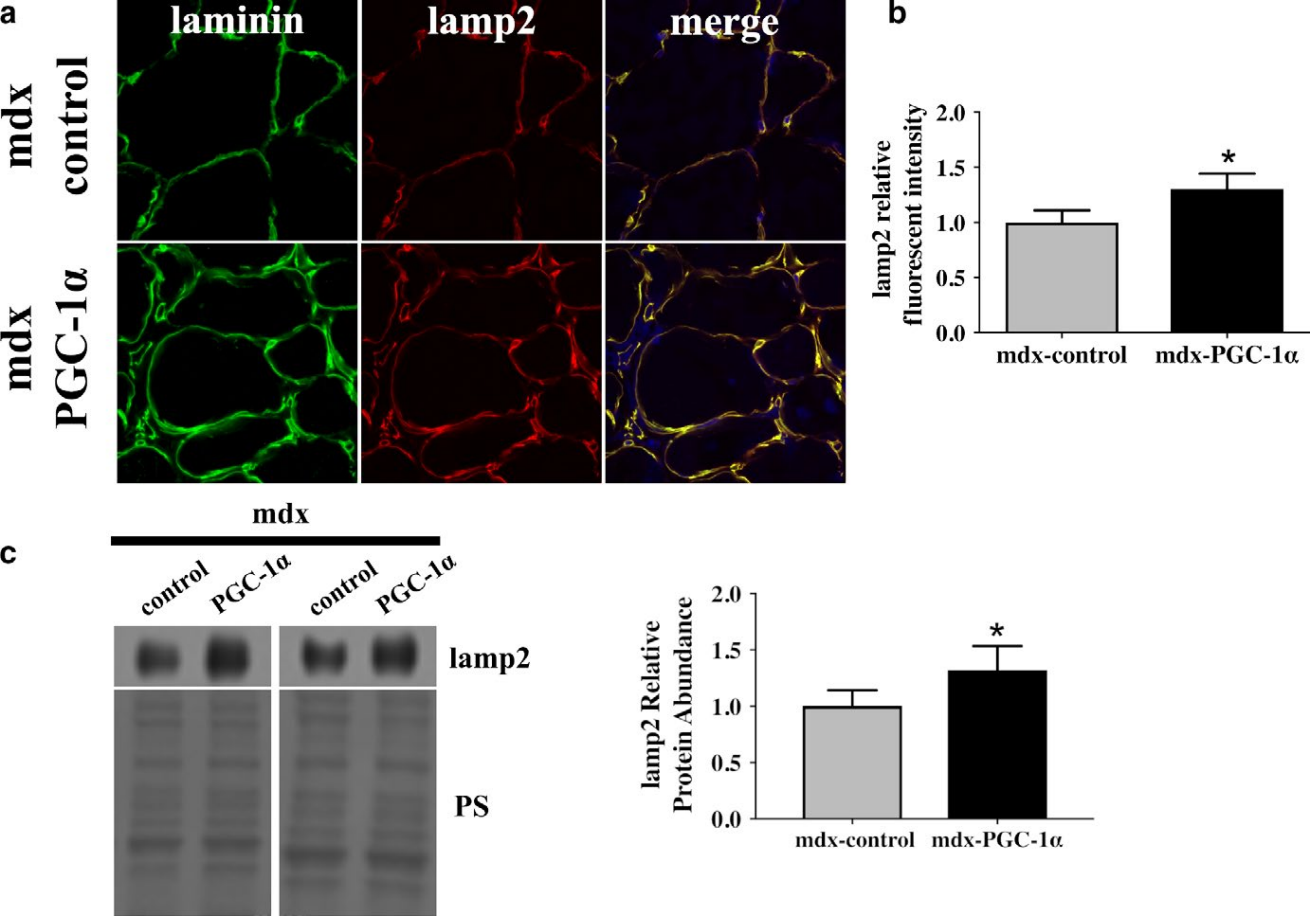
PGC‐1α overexpression increased lysosome abundance. (a) Mdx‐control and mdx‐PGC‐1α gastrocnemii were probed for lysosome marker, lamp2 (red), using immunofluorescence and nuclei (dapi, blue). (b) Quantification of lamp2 (red) fluorescent intensity, (*n* = 5/group). (c) Increased lamp2 was confirmed by Western blot, (*n* = 6/group). Ponceau S stain (PS) was used as a total protein loading control. * = significantly different from mdx‐control by paired sample *t* test, *p* < .05

**Figure 4:**
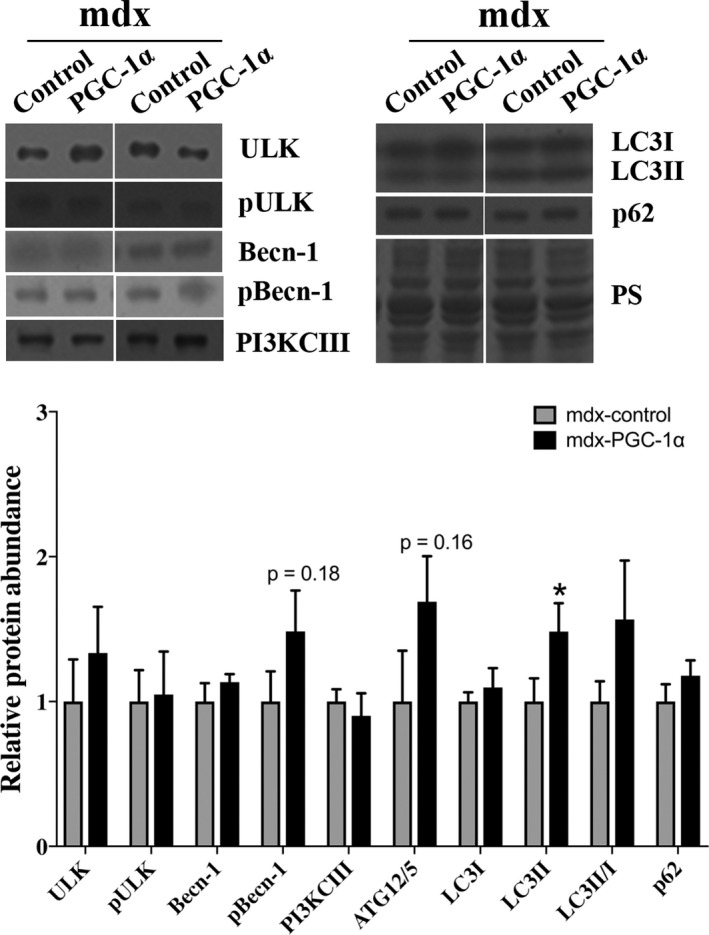
PGC‐1α overexpression increased autophagosome degradation. Markers of autophagy related proteins were largely unchanged with PGC‐1α overexpression. However, these data suggest increased autophagosome degradation due to increased LC3II with PGC‐1α overexpression and similar p62, an inverse correlate of autophagosome degradation. (*n* = 6/group). Ponceau S stain (PS) was used as a total protein loading control. * = significantly different from mdx‐control by paired sample *t* test, *p* < .05

Given that nuclear TFEB was elevated following PGC‐1α overexpression and that TFEB is a transcription factor that promotes lysosomal biogenesis, we next evaluated the effect of PGC‐1α overexpression on lysosome abundance. Using an immunofluorescent approach, we discovered that lamp2, a lysosomal marker, was increased by 30% (Figure 3a,b) in PGC‐1α‐overexpressing limbs compared to control limbs. These findings were confirmed by Western blot from whole homogenate (Figure 3c). As our working model is that degradation of autophagosomes in dystrophin‐deficient muscle is blunted, as least in part, due to a lysosomal insufficiency we further investigated the effect of increased lysosome abundance on markers of autophagy (Figure 4). Markers of autophagic activation, ULK and pULK (S555), and beclin‐1, a marker of autophagosome nucleation, were similar between mdx‐PGC‐1α and mdx‐control muscles. Both total beclin‐1 and p‐belin‐1 (S93) protein abundance were similar between groups despite the numerical elevation in p‐beclin (*p* = .18). Interestingly, PI3K CIII, a protein involved in autophagosome initiation, was similar between mdx‐control and mdx‐PGC‐1α as was Atg5/12 (*p* = .16). A marker of autophagosome formation, LC3II, was elevated with PGC‐1α overexpression, while p62, a marker of autophagosome degradation was similar between groups. Hence, elevated autophagosome formation paired with similar p62 abundance suggests increased degradation of autophagosomes following PGC‐1α overexpression.

## 4 DISCUSSION

Duchenne muscular dystrophy is a severe muscle wasting disease in which a host of cellular pathways are altered secondary to a dystrophin protein deficiency. We and others have recently established that autophagic dysfunction is included in the disease sequelae (Bibee et al., 2014; De Palma et al., 2012; Pal et al., 2014; Spaulding et al., 2018). More specifically, our data indicate lysosome abundance is decreased and autophagosome degradation is impaired in dystrophin‐deficient skeletal muscle (Spaulding et al., 2018). Previous evidence demonstrates that PGC‐1α overexpression decreased disease‐related injury and preserved muscle function using both prevention and rescue strategies (Chan et al., 2014; Handschin et al., 2007; Hollinger et al., 2013; Selsby et al., 2012). Emerging evidence points to a PGC‐1α‐mediated activation of TFEB (Erlich et al., 2018) while others have demonstrated a relationship between TFEB and lysosome biogenesis and autophagy (Sardiello et al., 2009; Settembre et al., 2011). We previously established that TFEB nuclear localization is decreased in dystrophin‐deficient skeletal muscle as was lysosomal abundance suggesting that decreased lysosomal content is due, at least in part, to dysregulation of TFEB (Spaulding et al., 2018). Further, as we and others have demonstrated decreased degradation of autophagosomes in dystrophin‐deficient muscle, this dysregulation of TFEB and subsequent blunting of lysosomal content may contribute to impaired degradation of autophagosomes in dystrophin‐deficient muscle (De Palma et al., 2012; Pal et al., 2014; Spaulding et al., 2018). Given this, we hypothesized that PGC‐1α overexpression in dystrophic muscle would drive TFEB nuclear translocation leading to increased lysosome abundance and increased degradation of autophagosomes.

While the underlying mechanism governing the relationship between PGC‐1α and TFEB is currently unknown, PGC‐1α appears to regulate expression of TFEB. For example, PGC‐1α overexpression or PGC‐1α knockout in healthy muscle results in a parallel effect on TFEB, such that PGC‐1α overexpression increased TFEB protein abundance (Vainshtein et al., 2015) and knockout decreased TFEB transcript and protein abundance (Erlich et al., 2018; Mansueto et al., 2017; Vainshtein et al., 2015). Given the relationship between PGC‐1α and TFEB and that our previous work hinted of a PGC‐1α‐mediated change in autophagy (Hollinger et al., 2013), continued study of tissues from 3 wk old mdx mice treated with PGC‐1α provided an opportunity for a probative study regarding TFEB and autophagy in dystrophin‐deficient muscle. Contrary to our expectations, in this investigation 3 wks following PGC‐1α gene transfer total TFEB abundance was similar between groups, however, nuclear TFEB was increased. These data indicate regulation of TFEB abundance and/or activity by PGC‐1α is more complex than simple transcriptional regulation and likely includes indirect regulation via post‐translational modification via a yet‐to‐be appreciated mechanism(s). Speculatively, as mTOR activity is known to be increased in dystrophin‐deficient muscle (Pal et al., 2014) and that phosphorylation of TFEB by mTOR, AKT or ERK1/2 leads to nuclear exclusion (Napolitano et al., 2018; Palmieri, Pal, & Sardiello, 2017; Settembre et al., 2011), mTOR‐mediated inhibition of TFEB and subsequently decreased lysosomal content seems likely. In support of this notion, PGC‐1α‐mediated inhibition of mTOR signaling has recently been demonstrated (Brown et al., 2018) providing a plausible and testable mechanism for PGC‐1α‐mediated regulation of TFEB localization. Moreover, we and others note smaller muscle masses in mice overexpressing PGC‐1α, which might be expected from mTOR inhibition (Hollinger et al., 2013; Hollinger & Selsby, 2015; Selsby et al., 2012).

PGC‐1α overexpression has been known for some time to preserve muscle function and decrease histopathological injury in dystrophin‐deficient muscle using prevention and rescue paradigms (Handschin et al., 2007; Hollinger et al., 2013; Hollinger & Selsby, 2015; Selsby et al., 2012). In addition to histological and functional protection in the dystrophin‐deficient soleus, PGC‐1α overexpression also altered gene expression suggestive of increased autophagy in the same animals used herein (Hollinger et al., 2013). In studies using limb muscle of approximately the same age, dystrophin deficiency has been shown to increase LC3I protein abundance while effects on LC3II are equivocal (Fiacco et al., 2016; Iannotti et al., 2019). In the present investigation evaluating the gastrocnemius, our data suggest increased autophagosome formation following PGC‐1α overexpression (increased LC3II) and increased degradation (similar p62). It seems likely this increased degradation was facilitated by increased lysosomal abundance. Further, given the relationship between PGC‐1α and TFEB as well as the role of TFEB in autophagy, mitophagy might also be improved following PGC‐1α overexpression. While mitophagy and mitochondrial biogenesis/content was beyond the scope of this investigation, mice treated with AICAR, which stimulates AMPK and ultimately PGC‐1α, increased BNIP3 protein abundance in dystrophin‐deficient skeletal muscle (Pauly et al., 2012) supporting the notion that PGC‐1α overexpression may stimulate mitophagy.

### 4.1 Perspectives and significance

These data indicate that PGC‐1α increased nuclear TFEB localization, lysosomal abundance, and degradation of autophagosomes. We speculate that increased PGC‐1α caused, directly or indirectly, increased nuclear TFEB translocation, which in turn, stimulated increased lysosome abundance, which allowed for greater degradation of autophagosomes. We temper our conclusions within the limitations of a probative study employing archived samples and encourage future investigations specifically intended to address questions raised herein. In addition to the role of TFEB‐mediated elevations in lysosome abundance, it is important to note that degradation of autophagosomes may be impacted by lysosome function (Gelman, Davis, Morris, & Gruenstein, 1981) and/or capacity for colocalization due to cytoskeletal derangement or impaired fusion. How these factors may interact and be altered by dystrophin deficiency remains largely unknown.

## CONFLICT OF INTEREST

MBH and JTS are cofounders of Extrave Bioscience, LLC. This relationship had no bearing on project objectives, data interpretation, or decision to publish.
